# Control of Complement Activation by the Long Pentraxin PTX3: Implications in Age-Related Macular Degeneration

**DOI:** 10.3389/fphar.2020.591908

**Published:** 2020-11-26

**Authors:** Matteo Stravalaci, Francesca Davi, Raffaella Parente, Marco Gobbi, Barbara Bottazzi, Alberto Mantovani, Anthony J. Day, Simon J. Clark, Mario R. Romano, Antonio Inforzato

**Affiliations:** ^1^ Department of Biomedical Sciences, Humanitas University, Milan, Italy; ^2^ Humanitas Clinical and Research Center IRCCS, Milan, Italy; ^3^ Istituto di Ricerche Farmacologiche Mario Negri IRCCS, Milan, Italy; ^4^ The William Harvey Research Institute, Queen Mary University of London, London, United Kingdom; ^5^ Wellcome Trust Centre for Cell-Matrix Research and Lydia Becker Institute of Immunology and Inflammation, Division of Cell-Matrix Biology and Regenerative Medicine, School of Biological Sciences, Faculty of Biology, Medicine and Health, University of Manchester, Manchester Academic Health Science Centre, Manchester, United Kingdom; ^6^ Universitäts-Augenklinik Tübingen, Eberhard Karls University of Tübingen, Tübingen, Germany; ^7^ The Lydia Becker Institute of Immunology and Inflammation, Faculty of Biology Medicine and Health, The University of Manchester, Manchester, United Kingdom; ^8^ Eye Center, Humanitas Gavazzeni-Castelli, Bergamo, Italy

**Keywords:** age-related macular degeneration, retinal pigmented epithelium, vitreous humor, complement system, alternative pathway, pentraxins, pentraxin 3

## Abstract

Dysregulation of the complement system is central to age-related macular degeneration (AMD), the leading cause of blindness in the developed world. Most of the genetic variation associated with AMD resides in complement genes, with the greatest risk associated with polymorphisms in the *complement factor H* (*CFH*) gene; factor H (FH) is the major inhibitor of the alternative pathway (AP) of complement that specifically targets C3b and the AP C3 convertase. Long pentraxin 3 (PTX3) is a soluble pattern recognition molecule that has been proposed to inhibit AP activation via recruitment of FH. Although present in the human retina, if and how PTX3 plays a role in AMD is still unclear. In this work we demonstrated the presence of PTX3 in the human vitreous and studied the PTX3-FH-C3b crosstalk and its effects on complement activation in a model of retinal pigment epithelium (RPE). RPE cells cultured in inflammatory AMD-like conditions overexpressed the PTX3 protein, and up-regulated AP activating genes. PTX3 bound RPE cells in a physiological setting, however this interaction was reduced in inflammatory conditions, whereby PTX3 had no complement-inhibiting activity on inflamed RPE. However, on non-cellular surfaces, PTX3 formed a stable ternary complex with FH and C3b that acted as a “hot spot” for complement inhibition. Our findings suggest a protective role for PTX3 in response to complement dysregulation in AMD and point to a novel mechanism of complement regulation by this pentraxin with potential implications in pathology and pharmacology of AMD.

## Introduction

Age-related macular degeneration (AMD) is a neurodegenerative and multifactorial disease of the eye that distinctively manifests itself with ageing (Jager et al., 2008), and is recognized as the leading cause of irreversible visual impairment in the elderly (Wong et al., 2014). Early and intermediate AMD is characterized by the accumulation of extracellular deposits (called drusen) between the retinal pigment epithelium (RPE) and the Bruch’s membrane (BrM). Eventually, the disease can progress either to dry AMD, with photoreceptor loss, or wet AMD, characterized by the formation of new blood vessels in the choroidal region that grow into the retina causing tissue disruption (Ambati et al., 2003).

Local dysregulation of the complement system and the associated chronic inflammation are major drivers of the disease (Ambati et al., 2013; Clark and Bishop, 2018). Complement proteins have been detected in the aqueous humor of AMD patients (Schick et al., 2017; Altay et al., 2019) and in drusen (Hageman et al., 2001; Crabb et al., 2002; Curcio, 2018). Moreover, most of the genetic variation underlying the risk of AMD resides in genes of the complement cascade (Fritsche et al., 2016; Cipriani et al., 2020). The rs1061170 single nucleotide polymorphism (SNP) in the *complement factor H* (*CFH*) gene (that leads to the Y402H amino acid substitution in the protein) has a strong association with the disease Edwards et al. (2005), Hageman et al. (2005), Haines et al. (2005). Factor H (FH) is the major soluble inhibitor of the alternative pathway (AP) of complement, and comprises 20 complement control protein (CCP) domains with distinct functions (Parente et al., 2017). CCPs1-4 mediate inhibition of assembly and stability of the AP C3 convertase (C3bBb), while CCPs6-8 and CCPs18-20 are involved in the recognition of “self” determinants (e.g., sulfated glycosaminoglycans, GAGs, and sialic acids) that are present on the surface of mammalian cells and extra-cellular matrices (ECMs), thus directing the inhibitory properties of FH towards host tissues (Clark et al., 2010; Clark et al., 2013; Blaum et al., 2015; Langford-Smith et al., 2015). The Y402H polymorphism (in CCP7) alters the binding of FH to heparan sulfate (in the BrM) (Parente et al., 2017; Clark and Bishop, 2018) and C reactive protein (CRP, which is present both in the BrM and choroid) (Sjoberg et al., 2007), thus leading to inappropriate complement activation and complement-driven inflammation, with pathological progression to AMD (Clark and Bishop, 2018).

One of the ligands of FH is the long pentraxin 3 (PTX3) (Deban et al., 2008), a multimeric glycoprotein with a distinctive quaternary structure (Bottazzi et al., 1997; Inforzato et al., 2008; Inforzato et al., 2010; Inforzato et al., 2012) that is produced by a number of somatic and immune cells, and plays key roles in innate immunity, tissue remodeling, and inflammation (Doni et al., 2019; Parente et al., 2019; Parente et al., 2020). Current evidence indicates that PTX3 is locally made by the RPE in the presence of pro-inflammatory cytokines (Woo et al., 2013), and in conditions of oxidative stress (Wang et al., 2016; Hwang et al., 2019). The protein has been detected in multiple layers of the human retina, including the BrM and the basement membrane of both RPE and choriocapillaris (Yamada et al., 2008). Furthermore, based on *in silico* analysis, *PTX3* transcription has been documented in the RPE/choroid region of the human eye, where it increases with age, although be it independently from the AMD status (Juel et al., 2015). Previously, we have reported that the PTX3 protein localizes underneath the BrM and in the choroid of both AMD and non-AMD donors (Swinkels et al., 2018), suggesting that PTX3 might act as a mediator of retinal homeostasis both in physiological and pathological conditions. Yet, the underlying mechanisms are still unclear. In this regard, in an animal model of AMD (induced by 4-hydroxynonenal, 4-HNE), genetic deficiency of *PTX3* led to enhanced complement activation, increased levels of C3a (a potent anaphylatoxin originating from proteolysis of C3) and the inflammatory cytokine IL-1β in the RPE, and accumulation of macrophages in the choroid (Wang et al., 2016), whereby PTX3 has been proposed to act as an anchoring molecule for FH in the BrM and RPE.

Here, we provided evidence that soluble PTX3 is present in the vitreous humor of both AMD and non-AMD human eyes. Using an established human RPE cell line (ARPE-19), we characterized the complement inhibiting-properties of PTX3 in physiological and inflammatory AMD-like (i.e., in the presence of TNF-α or IL-1β) conditions, and proposed a novel mechanism of complement regulation by this pentraxin with potential implications in the pathogenesis of AMD.

## Materials and Methods

### Donor Eye Tissue

Vitrous humor specimens were obtained from human eyes collected from the Manchester Royal Eye Hospital Eye Bank after removal of the corneas for transplantation. Consent had been obtained for the eye tissue to be used for research and guidelines established in the Human Tissue Act of 2004 (UK) were adhered to. Ethical approval for the use of human donor eyes was given by North West – Greater Manchester Central Research Ethics Committee (REC reference 15/NW/0932).

### Cell Cultures and Treatments

ARPE-19 cells (ATCC, CRL-2302) were cultured and stimulated as described in Supplementary Materials and Methods.

### Enzyme-Linked Immunosorbent Assays

The concentration of IL-6, IL-8 and VEGF in the cell culture medium was measured using the Quantikine ELISA assay (R&D Systems). The concentration of PTX3 both in the conditioned medium of ARPE-19 cells and in the vitreous humor of AMD and non-AMD donors was assessed using an in-house developed ELISA assay (Canovi et al., 2014).

### Binding Experiments

Binding of recombinant human PTX3 (rhPTX3) (Bottazzi et al., 1997) to ARPE-19 cells was assessed by flow cytometry. The interaction of C3b with rhPTX3 in the presence and absence of FH was assessed in solid phase and SPR binding experiments.

### Quantitative Real-Time Polymerase Chain Reaction

Total RNA was extracted from ARPE-19 cells using the Direct-zol^™^ RNA MiniPrep (Zymo Research) system, reverse trascribed to cDNA with the High Capacity cDNA Reverse Transcription Kit (Applied Biosystems), and amplified using the Sybr Green PCR Master Mix (Applied Biosystems). See Supplementary Table S1 for additional information.

### Western Blotting Analyses

Synthesis and secretion of the C3 and FB proteins in the conditioned medium of cultured ARPE-19 cells was assessed by western blotting by adaptation of a previous protocol (Baranova et al., 2014).

### Complement Activation Assays

Complement activation on plastic plate-immobilized and cell-bound proteins was evaluated by western blotting, ELISA and flow cytometry.

### Statistical Analyses

All data were analyzed using the Prism 8.0 Software (GraphPad Software).

See Supplementary Information for more details.

## Results

### Inflammatory Cytokines Induce Expression of Complement Activating Genes in ARPE-19 Cells

**FIGURE 1:**
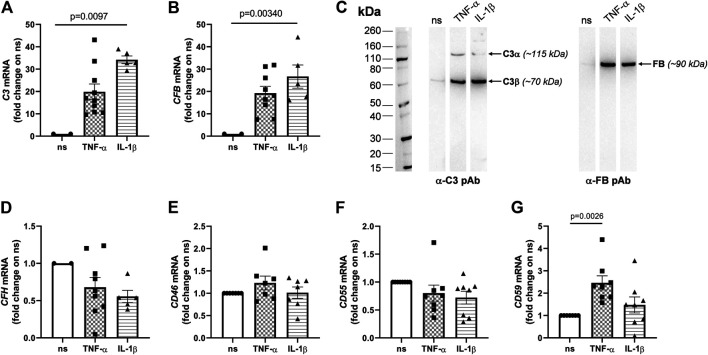
Pro-inflammatory cytokines induce transcription of the *C3* and *CFB* genes and secretion of the C3 and FB proteins in ARPE-19 cells. ARPE-19 were treated with 10 ng/ml TNF-α, 10 ng/ml IL-1β, or vehicle alone for 24 h, and RNA levels of **(A)** *C3*, **(B)** *CFB*, **(D)** complement factor H (*CFH*), **(E)** *CD46*, **(F)** *CD55*, and **(G)** *CD59* were measured by qRT-PCR. Data were normalized based on the mRNA levels from non-stimulated cells (ns; 2^−ΔCT *gene* − *GAPDH*^ values were in the range 0.00–0.04 for non-stimulated cells), and expressed as mean ± SEM (*n* = 5–10 from as many independent experiments). Statistical analysis was carried out using the Kruskall-Wallis test followed by Dunn’s multiple comparison test. **(C)** Presence of the C3 and FB proteins in cell culture media was assessed by western blotting. Proteins were separated by SDS-PAGE in reducing conditions, transferred onto PVDF membranes, and C3 (i.e., both α and β chains) and FB were revealed by appropriate polyclonal antibodies. Gels are shown that are representative of two independent experiments. Positions and apparent molecular weights of the C3α, C3β and FB bands are indicated.

Dysregulation of the complement system, in particular of the AP, is a critical factor in AMD pathogenesis (Clark and Bishop, 2018). We evaluated whether inflammatory AMD-like conditions (i.e., stimulation with TNF-α and IL-1β) could induce changes in the expression of soluble components of the AP and membrane-associated complement inhibitors in ARPE-19 cells (see Supplementary Table S1 for the sequence of the primers used in RT-PCR). The mRNA levels of both *C3* and *CFB* (components of the AP C3 convertase, C3bBb) significantly increased upon exposure to IL-1β, and showed an incremental trend following treatment with TNF-α (Figure 1A,B). Consistent with this, both cytokines induced synthesis and secretion of the C3 and FB proteins (Figure 1C). Furthermore, transcription of the *CFH* gene (Figure 1D) was somewhat (though not significantly) decreased, while *CD46* (also known as membrane cofactor protein, MCP, Figure 1E) and *CD55* (or decay-accelerating factor, DAF, Figure 1F) genes, encoding membrane associated AP inhibitors, did not change expression with the applied stimuli. Interestingly, *CD59* (or MAC-inhibitory protein, MAC-IP) mRNA (Figure 1G) was upregulated in ARPE-19 cells treated with TNF-α, but not IL-1β. These data indicate that inflammatory cytokines (particularly, IL-1β) induce an activation-prone profile in AP genes (i.e., increased levels of *C3* and *CFB* mRNAs and proteins) potentially leading to complement dysregulation in RPE cells. Moreover, when stimulated with IL-1β (and, to a lesser extent, TNF-α), these cells also synthesyzed and released pro-inflammatory (i.e., IL-6, IL-8) and pro-angiogenic (i.e., VEGF) factors (Supplementary Figure S1), which are involved in recruitment and activation of leukocytes (Tanaka et al., 2014; Harada et al., 1994; Shibuya, 2015), and might indirectly alter complement homeostasis in the RPE (Ambati et al., 2013; Luo et al., 2013).

#### PTX3 is Expressed by ARPE-19 Cells in Inflammatory Conditions and has No Direct Effect on Complement Activation on the Inflamed RPE

**FIGURE 2:**
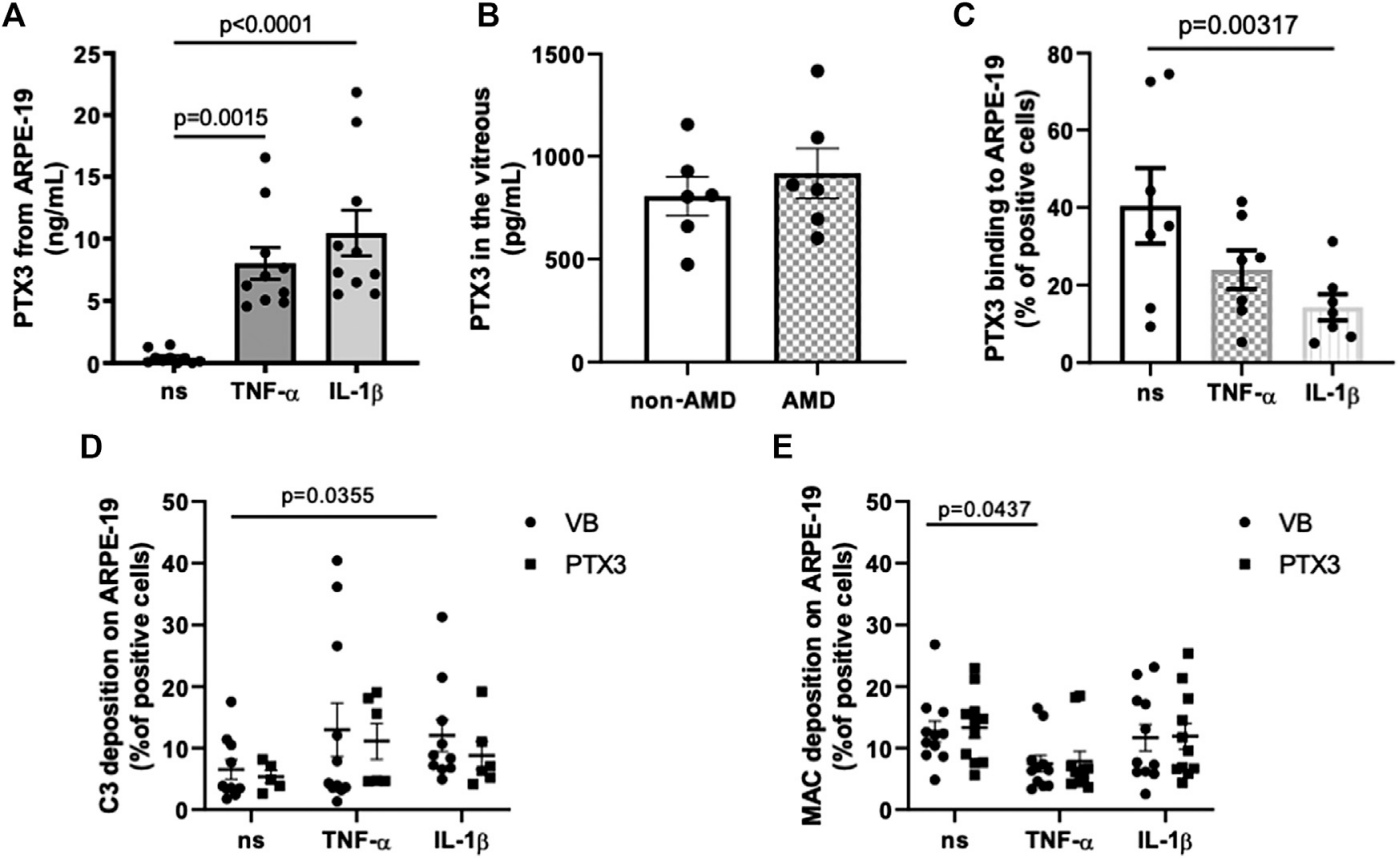
PTX3 is present *in vivo* in the human vitreous, binds *in vitro* ARPE-19 cells in non-inflammatory conditions, and has no direct effect on complement activation on IL-1β- or TNF-α-stimulated ARPE-19 cells. **(A)** ARPE-19 cells were treated with 10 ng/ml TNF-α, 10 ng/ml IL-1β, or vehicle alone for 24 h. PTX3 levels were measured in the conditioned medium. Data are expressed as mean ± SEM, n = 10. Friedman test followed by Dunn’s multiple comparison test. **(B)** Vitreous humor was collected from postmortem eyes of age-related macular degeneration (AMD) (*n* = 6) and non-AMD donors (*n* = 6) (see Supplementary Table S1), centrifuged for 15 min at 13,000 × g, and PTX3 levels were measured in the soluble fraction. Data are reported as mean ± SEM. **(C)** ARPE-19 cells were treated as described above and incubated with 10 µg/ml rhPTX3 for 30 min. Bound PTX3 was measured by flow cytometry (see Supplementary Figure S2 for a representative plot of event counts *vs* fluorescence intensity). Data are expressed as percentage of positive cells (mean ± SEM, *n* = 7). Analyzed by Kruskall-Wallis test followed by Dunn’s multiple comparison test. **(D,E)** ARPE-19 cells were stimulated with 10 ng/ml TNF-α or IL-1β for 24 h, followed by incubation with 10 µg/ml rhPTX3 (PTX3) or vehicle alone (Veronal Buffer, VB), and challenged with 10% normal human serum (NHS) for 30 min. Cell-bound C3 **(D)** and MAC **(E)** were measured by flow cytometry. Data are expressed as mean ± SEM, *n* = 9–11 from as many independent experiments. Analyzed by Kruskall-Wallis test, followed by Dunn’s multiple comparison test.

PTX3 is expressed by the RPE in the presence of pro-inflammatory cytokines (Woo et al., 2013). To model expression and function of PTX3 *in vitro* in inflammatory conditions, we treated ARPE-19 cells either with TNF-α or IL-1β, and measured the concentration of PTX3 in the culture medium (Figure 2A). Consistent with a previous report (Woo et al., 2013), both stimuli strongly induced synthesis and secretion of the protein (8.02 ± 1.27 ng/ml after TNF-α treatment, and 10.47 ± 1.83 ng/ml after IL-1β treatment, as compared to 0.43 ± 0.16 ng/ml in the absence of stimuli; mean ± SEM, n = 10). Moreover, we could detect endogenous PTX3 in the vitreous humor of a small cohort of 6 AMD and 6 non-AMD patients (Supplementary Table S2; Figure 2B). Similar protein levels were found in both groups with a trend towards increasing concentrations in AMD (805.2 ± 94.21 and 917.3 ± 121.1 pg/ml in non-AMD and AMD groups, respectively), indicating that soluble PTX3 (i.e., non-associated to the BrM) is present in the human eye both in physiological and pathological conditions.

PTX3 binds apoptotic cells and modulates complement deposition on these cells through engagement of FH (Deban et al., 2008) and C4b-binding protein (C4BP, Braunschweig and Jozsi, 2011). Furthermore, genetic deficiency of PTX3 is associated with increased deposition of C3 on sarcoma cells, and administration of the exogenous protein abrogates this effect via FH-dependent mechanisms (Bonavita et al., 2015). We therefore questioned if the secreted protein could bind the RPE cell line in basal and inflammatory (when complement activating genes are up-regulated) conditions. To this end, 10 µg/ml recombinant human PTX3 were added to ARPE-19 cells that had been previously treated with TNF-α or IL-1β, and the protein’s binding was analyzed by flow cytometry (Figure 2C; Supplementary Figure S2). PTX3 bound these cells in all conditions, however with reduced strength following IL-1β stimulation. These findings extend the current view that inflammation up-regulates the synthesis of PTX3 in the RPE, and suggest that the recruitment of PTX3 onto the RPE is inhibited by the same inflammatory cytokines that promote its expression (particularly, IL-1β).

We then investigated the effect of RPE-bound PTX3 on complement activation. To this end, ARPE-19 cells stimulated either with TNF-α or IL-1β and pre-incubated with rhPTX3, were challenged with normal human serum (NHS), and complement activation was assessed by measuring cell-bound C3 and MAC by flow cytometry. Treatment with IL-1β led to increased deposition of C3 (Figure 2D), consistent with the effect of this cytokine on *C3* and *CFB* expression (Figures 1A–C), however it did not affect MAC formation (Figure 2E). Furthermore, TNF-α-stimulated ARPE-19 cells had reduced assembly of MAC (compared to non-stimulated cells, Figure 2E) and similar C3 deposition (Figure 2D), consistent with *CD59* (inhibitor of MAC) being up-regulated by TNF-α (Figure 1G). More importantly, pre-incubation of cells with PTX3 did not change the levels of C3 (and MAC) following IL-1β (and TNF-α) stimulation (Figures 2D,E), consistent with reduced protein’s binding (Figure 2C). Furthermore, non-stimulated ARPE-19 cells had baseline levels of C3 and MAC deposition, which were not further reduced by PTX3. These findings suggest that inflammatory stimuli (mainly IL-1β) promote complement activation on the RPE, and PTX3 could not control this process due to an impairement of its interaction with the “inflamed” RPE.

### Pentraxin 3 Modulates Alternative Pathway Activation and Membrane Attack Complex Formation on Non-Cellular Surfaces via Recruitment of C3b and Factor H

**FIGURE 3:**
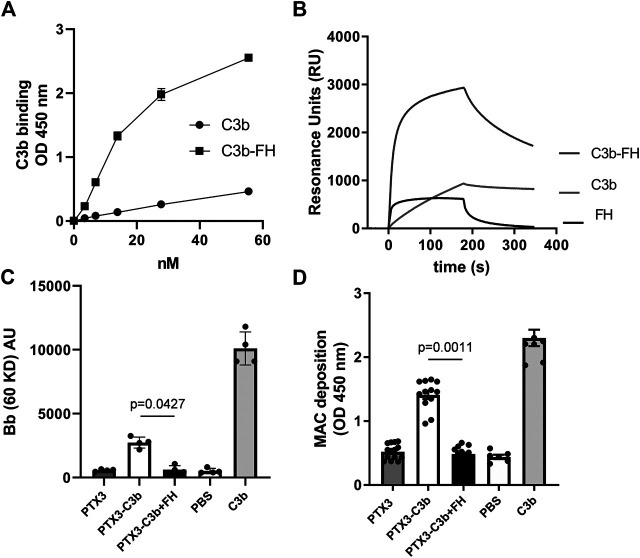
PTX3 recruits C3b and FH on non-cellular surfaces and modulates C3bBb and MAC formation. **(A)** PTX3-coated wells were incubated with the indicated concentrations of C3b or mixtures of C3b and FH (at [C3b]:[FH] molar ratios of 2:1). Bound C3b was detected by ELISA, and data are presented as mean ± SEM from two independent experiments performed in triplicate (*n* = 6). **(B)** Representative SPR sensorgrams of the interaction of FH, C3b, and mixtures of C3b and FH with immobilized PTX3. **(C)** Microtiter plates were coated with C3b alone, PTX3 followed by C3b, or PTX3 followed by a mixture of C3b and FH, then incubated with FH-depleted human serum. Surface-bound proteins were analyzed by western blotting. Intact factor B and its proteolytic fragment Bb (indicative of C3bBb formation) were revealed as immune-reactive bands at apparent molecular weights of 93 kDa and 60 kDa. Band intensity for the Bb species was measured by densitometry. Results are expressed as mean ± SEM, *n* = 4. **(D)** Microtiter plates were coated and incubated as described in **(C)**, and MAC deposition was assessed by ELISA using an anti-sC5b-9 antibody. Data are expressed as mean ± SEM from four independent experiments performed in duplicate (*n* = 8). The p-values reported in **(C,D)** were from the Kruskall-Wallis test, followed by Dunn’s multiple comparison test, for the PTX3/C3b and PTX3/C3b/FH complexes. Both in **(C,D)**, C3b-coated wells were used as a positive control for C3bBb formation, and not used for statistical comparison.

Following our observation that this pentraxin is not involved in the regulation of complement activation on ARPE-19 cells (stimulated with inflammatory cytokines), we investigated the mechanisms by which it modulates complement on non-cellular surfaces, using microtiter plastic plates as an *in vitro* model. To this end, we analyzed the binding of C3b to PTX3 in the presence or absence of FH (i.e., at a [C3b]:[FH] molar ratio of 2:1, which approximates that of [C3]:[FH] in the human plasma (Merle et al., 2015)), and observed that PTX3 recognized C3b, and the resulting complex was strenghtened by FH (Figure 3A). These interactions were further evaluated by SPR. C3b stably bound PTX3, due to slow dissociation. Conversely, FH rapidly associated to and dissociated from surface-captured PTX3, indicating a more dynamic interaction. However, when a mixture of C3b and FH was injected over PTX3-coated sensorchips, strong binding signals were observed with complex (non-Langmuir) kinetics (Figure 3B), indicating that surface-bound PTX3 recruits C3b with high affinity through formation of a stable ternary complex mediated by FH.

We then questioned whether the PTX3-bound C3b (either in the presence or in the absence of FH) retained the ability to activate complement. To this aim, microtiter plates were coated with PTX3 followed by either C3b or a mixture of C3b and FH, and incubated with FH-depleted human serum (FHDHS, to model complement dysregulation). The resulting protein complexes were retrieved from wells and analyzed by WB, where Bb bands are indicative of C3bBb convertase formation and AP activation (Hourcade, 2006) (see a representative gel in Supplementary Figure S3, and the combined data in Figure 3C). PTX3-bound C3b formed active C3bBb convertase, although to a lesser extent than C3b alone [possibly due to differences in the relative amounts of plastic-absorbed and PTX3-bound C3b, and to fluid phase C3b being unable to form covalently bound AP C3 convertase (Merle et al., 2015)] (lane 6 in Supplementary Figure S3, and Figure 3C). However, when FHDHS was added to PTX3-coated wells that had been incubated with a mixture of C3b and FH, no Bb band was observed, indicating that PTX3-and FH-bound C3b cannot form active C3bBb convertase (lane 7 in Supplementary Figure S3, and Figure 3C). No signal was recorded when PTX3-coated wells were incubated with FHDHS (lane 5 in Supplementary Figure S3). Formation of the MAC complex, terminal pathway of the complement cascade (Merle et al., 2015), was also investigated. Similar to C3bBb deposition, C3b supported MAC formation when in a binary complex with PTX3 but failed to do so when incubated both with PTX3 and FH (Figure 3D). In additional experiments, fluid phase PTX3 retained binding to C3b-coated plates, and this interaction was enhanced by FH, although to a minor extent compared to surface-bound PTX3 (Supplementary Figures S4A and, for comparison, Figure 3A). Furthermore, PTX3-bound C3b formed active C3bBb (similar to C3b alone), and lost this function when FH was added to the system (i.e. forming a C3b/PTX3/FH complex) (Supplementary Figures S4B,C).

## Discussion

An age-related decline in the physiological function of the RPE and subsequent alterations in immune homeostasis within and around the outer blood-retinal barrier contribute to the chronic inflammation that underlies AMD pathogenesis (Ambati et al., 2013). Complement dysregulation and the associated complement-driven inflammation are key events in the onset and progression of this disease (Clark and Bishop, 2018; Pauly et al., 2019). Complement effectors, such as C3a, C5a and the membrane attack complex (MAC), can induce the expression of pro-inflammatory cytokines (including TNF-α and IL-1β) and growth factors (e.g., VEGF) in the RPE (Lueck et al., 2011; Wang et al., 2016), a process that has been associated with AMD progression (Natoli et al., 2017b; Touhami et al., 2018). Moreover, up-regulation of C3 in microglia cells has been linked to retinal damage in AMD (Rutar et al., 2014; Natoli et al., 2017a). Here, we report that, when treated with IL-1β (and TNF-α), ARPE-19 cells (an *in vitro* model of the human RPE) up-regulated transcription of the *C3* and *CFB*, but not *CFH*, genes, and synthesis/secretion of the C3 and FB proteins, which is indicative of complement dysregulation in the inflamed RPE. Similar profiles have been reported in ARPE-19 cells cultured in the presence of conditioned medium from activated microglia (Madeira et al., 2018) and *in vitro* polarized macrophages (Luo et al., 2013). In addition, treatment of ARPE-19 cells with TNF-α promoted expression of the MAC inhibitor CD59, potentially contributing to the control of complement-driven inflammation. In this regard, decreased levels of CD59 have been associated with AMD pathology (Lueck et al., 2011; Ebrahimi et al., 2013). Consistent with the observed gene expression profiles, IL-1β increased susceptibility of ARPE-19 cells to C3 deposition, whereas stimulation with TNF-α led to reduced MAC formation on the cell surface. These findings point to a complex relationship between inflammatory cytokines and complement activation/regulation in the RPE.

A close crosstalk between the long pentraxin PTX3 and the complement system has been described in diverse tissues and conditions, which regulates complement-dependent inflammatory responses. Current literature indicates that PTX3 is made locally by the RPE in conditions of inflammation (Woo et al., 2013) and oxidative stress (Wang et al., 2016; Hwang et al., 2019), and localizes in the BrM and the basement membrane of both RPE and choriocapillaris, concentrating in the intercapilary septa (Yamada et al., 2008; Swinkels et al., 2018). In agreement with previous evidence (Woo et al., 2013), here we report that ARPE-19 cells express high levels of this pentraxin, when exposed to TNF-α and IL-1β. Presence of the PTX3 protein in the RPE/BrM/choroid has been documented both in AMD (Yamada et al., 2008) and, more recently, in tissues from non-AMD donors (Swinkles et al., 2018). In the present study, we found soluble PTX3 protein in the humor vitreous of both AMD and non-AMD patients, consistent with the view that this pentraxin is constitutively expressed in the eye, and likely contributes to local tissue homeostasis. A trend of increasing concentrations of PTX3 in the AMD vitreous was observed (similar to that we have described in the choriocapillaris (Swinkels et al., 2018)), which however was not significant, likely due to the small sample size and the analyzed AMD specimens all being from donors in the early stage of the disease. At present, it is not clear if other cell types of the human eye, in addition to the RPE (and choroid), can synthesize the protein. Given that PTX3 expression has been consistently documented in human leukocytes (Doni et al., 2019), it is plausible that this pentraxin is additionally made and released by vitreous body-resident phagocytes (and possibly, retinal microglia and Müller cells), contributing to complement homeostasis in the eye (Pauly et al., 2019).

PTX3 binds several complement proteins, including C1q, mannose binding lectin, ficolin-1 and -2, and activates the classical and lectin pathways (Bottazzi et al., 2010; Bally et al., 2019; Parente et al., 2020). In addition to activators, PTX3 interacts with complement inhibitors like FH and C4BP (Deban et al., 2008; Braunschweig and Jozsi, 2011). Based on an animal model of oxidative stress-induced AMD, PTX3 has been proposed to inhibit AP activation (and the subsequent complement-dependent inflammation) via recruitment of FH (Wang et al., 2016). Furthermore, PTX3 has been described to bind apoptotic and cancer cells and modulate complement activation on these cells through engagement of soluble complement inhibitors (Deban et al., 2008; Braunschweig and Jozsi, 2011; Bonavita et al., 2015). Based on this rationale, here we investigated the AP/PTX3 crosstalk in three different settings (i.e., cell-bound, surface-immobilized and fluid phase PTX3) that recapitulate biologically relevant ways of the presentation of this long pentraxin in the eye.

First, we observed that PTX3 bound ARPE-19 cells in physiological (non-inflammatory) conditions, and this interaction was significantly reduced when these cells were treated with IL-1β, but not TNF-α. Arguably, IL-1β induces modifications in structure/composition of the cell membrane that affect its binding to PTX3. In this regard, we observed that IL-1β prompted synthesis and secretion of IL-6, IL-8 and VEGF by ARPE-19 cells, thus establishing a “self-sustained” inflammatory status that likely modifies plasma membrane and membrane receptor dynamics (Kauppinen et al., 2016). Consistent with the observed binding properties of PTX3, this pentraxin could not protect IL-1β-treated ARPE-19 cells (that were more susceptible to C3 deposition) from complement activation. It is worth pointing out here that whole human serum was used in these experiments as a source of complement, which does not necessarily recapitulate composition and concentration of this system in the eye (Clark and Bishop, 2018; Pauly et al., 2019). Furthermore, the ARPE-19 cells were cultured in non-polarizing conditions, which raises the possibility that either the apical or basal surface (or both) of these cells might retain binding to PTX3 when exposed to inflammatory cytokines. These limitations notwithstanding, it is conceivable that the complement regulating functions of PTX3 mainly involve extracellular matrix (ECM) compartments of the eye, including the BrM, choroidal septa and, based on our evidence, vitreous body, where the protein has been localized.

**FIGURE 4:**
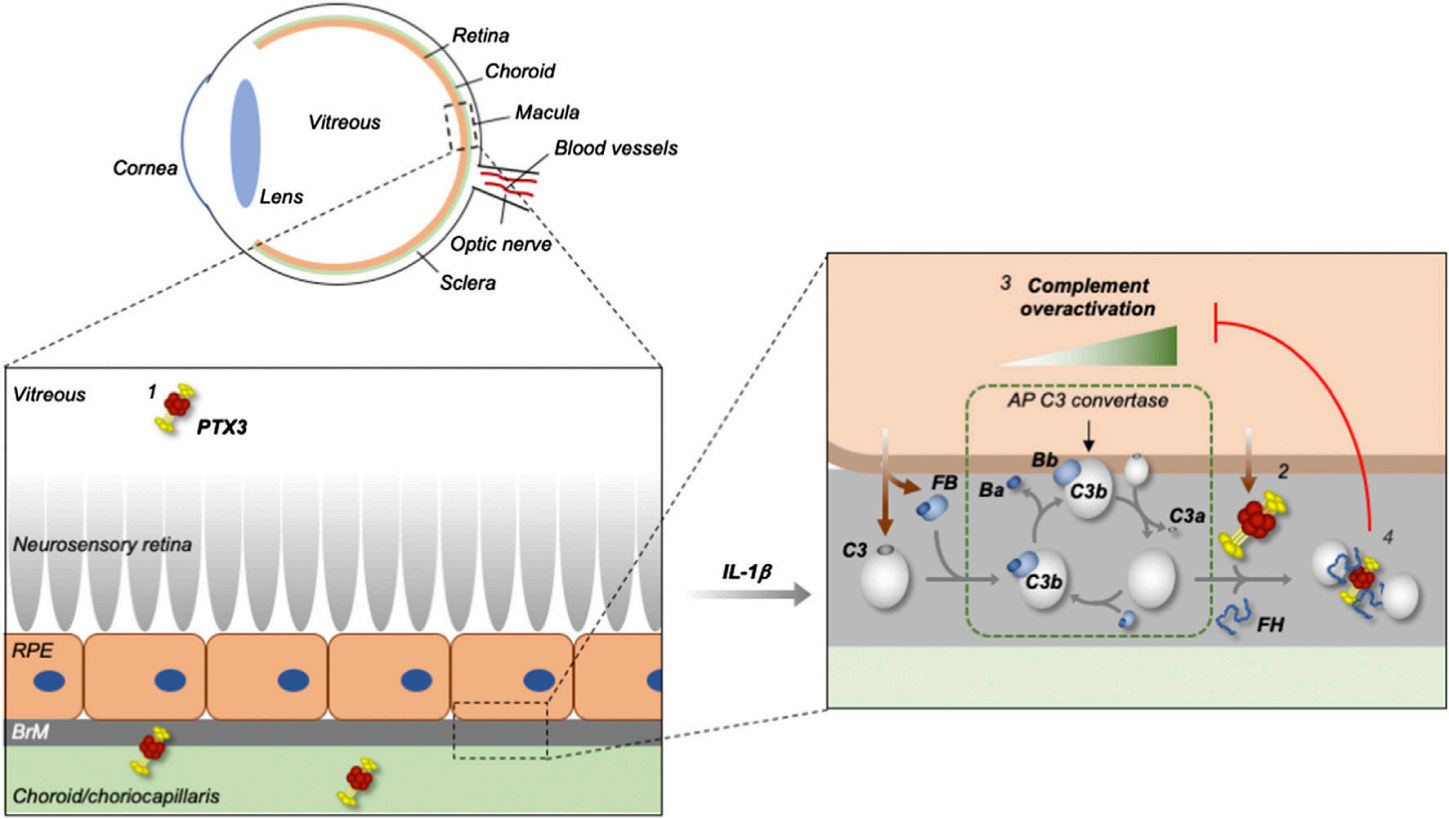
Proposed mechanism for the control of complement dysregulation by PTX3 in the human eye. PTX3 is present in the human vitreous (in addition to the retinal pigment epithelium (RPE) and choroid) both in physiological and pathological (AMD) settings (1), and overexpressed by the RPE (modelled *in vitro* using ARPE-19 cells) in inflammatory conditions (2) that set the scene for complement (mainly AP) dysregulation (3). The secreted PTX3 protein has impaired binding to the inflamed RPE, and thus fails to protect it from complement activation, however it inhibits the AP on non-cellular surfaces (likely the extra-cellular matrix (ECM) of the Bruch’s membrane (BrM) and choroid/choriocapillaris, as exemplified in the scheme), through formation of a stable PTX3/FH/C3b complex that acts as a “trap” for C3b and “hot spot” for complement inhibition (4).

We then investigated the mechanisms underlying the PTX3/complement crosstalk in a cell-free setting. Surface-absorbed and fluid phase PTX3 was able to bind C3b, key activator of the AP, and this association was strengthened by FH. To our knowledge, this is the first report of a direct interaction between PTX3 and C3b. Interestingly, in SPR experiments we found that immobilized PTX3 formed a ternary complex with FH and C3b with a distinctive association/dissociation profile as compared to that of individual FH and C3b. Detailed analysis of the underlying kinetics and binding mechanisms was beyond the scope of this study, however, it is worth noting here that this complex was long-lived in the applied conditions, with remarkably fast association and slow dissociation rates. To assess if C3b in the PTX3/C3b/FH complex retained complement-activating functions, we developed an *in vitro* assay where microtiter plates were coated with PTX3/C3b or PTX3/C3b/FH complexes, and incubated with human serum that lacked FH (to mimic AP dysregulation). These experiments demonstrated that C3b supports AP activation and MAC formation when bound to PTX3 (in a PTX3/C3b binary complex), and loses these functions when additionally associated with FH (in a PTX3/C3b/FH ternary complex), indicating that in the applied setting inhibition of complement activation is mediated by FH binding to C3b. Based on these findings, we propose that PTX3 cooperates with FH to form a “molecular trap” for C3b, which, when engaged by PTX3 and FH, is no longer accessible to FB and cannot activate the AP and amplify the complement cascade (see Figure 4). This mechanism is mostly apparent when PTX3 is bound to non-cellular surfaces (in a setting that mimics the ECM-attached protein) in the presence of soluble C3b. In these conditions, C3b likely has its activated thioester hydrolysed and cannot form covalently bound AP C3 convertase. However, it can still assemble the convertase in solution, and support generation of the anaphylatoxin C3a (Merle et al., 2015). It is therefore conceivable that the C3b-trapping properties of PTX3 (in concert with FH) reduce the intra-ocular levels of C3a (and, possibly, C3a-induced inflamatory mediators) as previously suggested by experimental modelling of AMD in *Ptx3*-deficient mice (Wang et al., 2016).

We found that, in response to inflammatory stimuli, ARPE-19 cells up-regulated synthesis and secretion of both PTX3 (AP inhibitor) and C3 and FB (AP C3 convertase components) proteins, pointing to a compensatory role for this pentraxin in inflammation. This mechanism could be relevant in the presence of the AMD-associated 402H variant of FH, which has been demonstrated to have a more restricted specificity for sulfated GAGs compared to 402Y (Clark et al., 2006; Prosser et al., 2007), and likely has decreased ability to control complement activation at ECM sites, such as the BrM (Clark et al., 2010; Clark and Bishop, 2018). In line with this hypothesis, PTX3 has been shown to co-localize with FH in the murine inner BrM and RPE (Wang et al., 2016). Also, we have described that the Y402H polymorphism alters the binding of FHL-1 (a truncated form of FH), but not FH, to PTX3 (Swinkles et al., 2018). FHL-1 predominates over FH in the BrM and choroid (Clark et al., 2014), and shares with PTX3 common sites within these regions (Swinkels et al., 2018). Thus, it is plausible that the interaction between PTX3 and FHL-1 (in addition to FH) control availability and function of this complement inhibitor (and FH).

In summary, our findings suggest a protective and compensatory role for PTX3 in response to complement dysregulation in AMD, and point to this pentraxin as a potential candidate for novel pharmacological treatments of the disease.

## Data Availability Statement

The raw data supporting the conclusions of this article will be made available by the authors, without undue reservation.

## Ethics Statement

The studies involving human participants were reviewed and approved by North West – Greater Manchester Central Research Ethics Committee (REC reference 15/NW/0932). The patients/participants provided their written informed consent to participate in this study.

## Author Contributions

MS designed and conducted all the experimental activities on this study and wrote the manuscript. FD, RP, and MG assisted on complement deposition, flow cytometry and SPR analyses, respectively. BB, AM, SJC, AJD, and MRR critically revised the manuscript. AI supervised the study and wrote the manuscript.

## Funding

MRR is recipient of a Research Prize from the Italian Society of Ophtalmology (SOI) that funded FD. The financial support of Fondazione Beppe and Nuccy Angiolini to RP is greatly acknowledged.

## Conflict of Interest

AJD is a co-Founder and Director of Link Biologics. SJC is a co-Founder and Director of Complement Therapeutics.

The remaining authors declare that the research was conducted in the absence of any commercial or financial relationships that could be construed as a potential conflict of interest.

The handling editor declared a past co-authorship with one of the authors MRR.
