# Supplementary material for: Control of Complement Activation by the Long Pentraxin PTX3: Implications in Age-Related Macular Degeneration
Source: Front Pharmacol. 2020 Nov 26;11:591908. doi: 10.3389/fphar.2020.591908 (PMC7725797; doi:10.3389/fphar.2020.591908)
Supplement: Supplementary file 1 [file datasheet1.docx]

Control of complement activation by the long pentraxin PTX3: implications in age-related macular degeneration

*Matteo Stravalaci, Francesca Davi, Raffaella Parente, Marco Gobbi, Barbara Bottazzi, Alberto Mantovani, Anthony J. Day, Simon J. Clark, Mario R. Romano, Antonio Inforzato*

**Supplementary Materials and Methods**

**Donor Eye Tissues**

Post-mortem donor eyes were obtained from the Manchester Eye Bank at the Royal Eye Hospital (Manchester, UK), within 48 hours from the time of death; these were classified and curated as part of the Manchester Eye Tissue Repository (ETR). No organs/tissues were procured from prisoners. Our research adhered to the tenets of the Declaration of Helsinki and in all cases, there was prior informed consent for the eye tissue to be used for research obtained and held by the Manchester Eye Bank, and guidelines established in the Human Tissue Act of 2004 (UK) were followed. Ethical approval for the use of tissue in these experiments was granted by the Manchester Eye Tissue Repository ethics committee (ref.15/NW/0932). Except in the case of AMD tissues, none of the other donors had a history of visual impairment or eye disease; see Supplementary Table 1. Post mortem times for non-AMD and AMD donor tissue were similar with an average of 45 hours (range 39–48 h) and an average 37 hours (range 32–43 h), respectively. Vitreous humor was withdrawn by syringe aspiration from the center of the eyeball, and stored at −80°C as previously described (Angi et al., 2012). All donor eyes were classified as early AMD or non-AMD based on the presence of clinically sized drusen in the macula as observed by post-mortem fundus imaging, which is carried out routinely within the Manchester ETR.

**Cell cultures and treatments**

ARPE-19 cells (ATCC, CRL-2302) were cultured in high glucose Dulbecco’s Modified Eagle’s Medium (DMEM) supplemented with 10% (v/v) Fetal Bovine Serum (FBS), L-Glutamine and 1% penicllin/streptomycin, at 37°C in a humidified atmosphere of 5% CO_2_. Cells were plated at a density of 3x10^6^ cells/well on 6-well plates, prior to incubation with either 10 ng/mL recombinant TNF-α or 10 ng/mL recombinant IL-1β (Peprotech, UK) for 24 hours at 37°C. Control cells were incubated with DMEM only.

**Binding of PTX3 to ARPE-19 cells**

ARPE-19 cells, cultured and treated as described above, were detached by incubation with Accutase (Thermo Fisher Scientific, USA). 2x10^5^ cells (per condition) were incubated with 10 µg/mL recombinant human PTX3 (Bottazzi et al., 1997) in PBS containing 0.90 mM CaCl_2_, 0.50 mM MgCl_2_ and 2% (w/v) BSA (washing buffer) for 30 minutes at room temperature. Cells were washed twice with washing buffer, and further incubated for 1 hour at room temperature with 0.5 µg/mL of an in-house developed rabbit anti-human PTX3 polyclonal antibody (Bottazzi et., 1997), followed by incubation with Alexa 647-conjugated donkey anti-rabbit IgG polyclonal antibody (2 µg/mL, Thermo Fisher Scientific). Following additional washings, cells were fixed with 1% (w/v) paraformaldehyde (PFA). Cell-bound PTX3 was detected by Flow Cytometry on a FACSCanto II system (Beckman Dickinson Biosciences, USA), and data were analyzed with the FlowJo software (Beckman Dickinson).

**Quantitative Real-Time Polymerase Chain Reaction (qRT-PCR)**

Total RNA was extracted from ARPE-19 cells using the Direct-zol™ RNA MiniPrep Kit (Zymo Research, USA), according to the manufacturer’s instructions. RNA was quantitated by UV absorbance using a NanoDrop 2000 system (Thermo Fisher Scientific), and stored at −80°C. 1 µg of total RNA (per condition) was reverse-transcribed to cDNA using the High Capacity cDNA Reverse Transcription Kit (Applied Biosystems, USA), according to the instructions provided by the manufacturer. 50 ng of cDNA (per condition) were amplified with Sybr Green PCR Master Mix (Applied Biosystems) and 500 nM primers (10 μl final volume), using a Quantiflex Studio 7 apparatus (Applied Biosystems). The sequences of the primers used in these experiments are provided in Table S1. Reaction parameters were as follows: 20 seconds 95°C hold, followed by 40 cycles of 1 second-long 95°C melt and 20 seconds-long 60°C anneal/extension. Ct values were expressed as “relative quantification” using the 2∆∆Ct method, according to the method described by Livak et al. (Livak and Schmittgen, 2001). *GAPDH* was used as housekeeping gene.

**Western blotting analysis of cell culture media**

To assess synthesis and secretion of the C3 and FB proteins, ARPE-19 cells were cultured as described above, and incubated with either 10 ng/mL recombinant TNF-α or 10 ng/mL recombinant IL-1β in DMEM without FCS for 24 hours at 37°C. Control cells were incubated with DMEM in the absence of stimuli. Total proteins in cell culture media were concentrated and analyzed by western blotting by adaptation of a previous protocol (Baranova et al., 2014). Briefly, cell culture media (1 ml per condition) were incubated with 25 μl (packed resin volume) of StrataClean resin (Agilent Technologies, USA) for 15 min at room temperature under agitation, followed by a water wash. The resin-bound material was denatured and reduced by heating at 70 °C for 10 min in sample loading buffer containing DTT (Life Technologies, USA). Proteins were separated by SDS-PAGE on 4-12% BisTris gels (Life Technologies), using MOPS SDS running buffer, and transferred onto 0.45-μm PVDF membranes. C3 (i.e., both α and β chains) and FB were revealed with a goat anti-human C3 polyclonal antibody (1 in 5,000 dilution; Merck Millipore, USA) and a goat anti-human FB polyclonal antibody (1 in 2,000 dilution; Quidel, USA), respectively, followed by an HRP-conjugated donkey anti-goat IgG polyclonal antibody (1 in 5,000 dilution, R&D Systems). Chemiluminescence was recorded on a Chemidoc system (Bio-Rad Laboratories, USA), following the addition of enhanced chemiluminescence substrate (Merck Millipore).

**Solid Phase Binding Assays**

rhPTX3 (10 µg/well in PBS) and purified human C3b (3 µg/well in PBS; Complement Technology, USA) were absorbed overnight at 4°C onto Nunc-Immuno MaxiSorp 96-well plates (Corning, USA). PBS only was used for blank wells, and plates were washed three times with PBS containing 0.90 mM CaCl_2_, 0.50 mM MgCl_2_ and 0.1% (v/v) Tween-20 at pH 7.0 (washing buffer) throughout the assay. Following saturation of uncoated sites with 1% (w/v) BSA (Sigma Aldrich, USA) in washing buffer for 1 hour at 37°C, plates were incubated with either C3b (for PTX3-coated wells) or rhPTX3 (for C3b-coated wells) in the presence or absence of purified FH (Complement Technology, USA) for 1 hour at 37°C. FH was applied at [C3b]:[FH] and [PTX3]:[FH] molar ratios of 2:1. Bound C3b was revealed with a goat anti-human C3 polyclonal antibody (1 in 5,000 dilution), followed by an HRP-conjugated donkey anti-goat IgG polyclonal antibody (1 in 5,000 dilution). Bound rhPTX3 was revealed with a rabbit anti-human PTX3 polyclonal antibody (1 in 5,000 dilution), followed by an HRP-conjugated donkey anti-rabbit IgG polyclonal antibody (1 in 5,000 dilution; GE Healthcare, USA). The chromogenic substrate 3,3',5,5'-tetramethylbenzidine (TMB, Thermo Fisher Scientific) was added, and the colorimetric reaction was stopped with 2N H_2_SO_4_. Absorbance was read at 450 nm on a Spectrostar Nano Microplate Reader (BMG Labtech, Germany). Values from blank wells were subtracted from those recorded at sample wells.

**Surface Plasmon Resonance (SPR)**

SPR analyses were carried out using a Proteon XPR36 system (Bio-Rad Laboratories), which has six parallel flow channels that can immobilize up to six ligands on the sensor chip surface. After ligand immobilization, the ProteOn XPR36 fluidic system can rotate by 90° (Bravman et al., 2006), so that up to six different analytes can be injected simultaneously over the ligands. A rabbit anti-human PTX3 polyclonal antibody was immobilized on both sample and reference channels of GLC sensor chips (Bio-Rad Laboratories) by amine coupling chemistry, as previously described (Canovi et al., 2014). 30 µg/mL of rhPTX3 in PBS were then flowed over the immobilized antibody in the sample channel until saturation of the binding signal. Bound PTX3 levels were about 2000 resonance units (RU, where 1 RU = 1 pg protein/mm^2^). Buffer only (i.e., PBS) was injected into the reference channel. After rotation of the microfluidic system, purified C3b, FH or a mixture of both in a PBS solution containing 0.90 mM CaCl_2_ and 0.50 mM MgCl_2_ plus 0.005% (v/v) Tween-20 at pH 7.0 (running buffer) were injected simultaneously into both sample and reference channels for 3 minutes, and dissociation was followed for 3 minutes. Parallel injections of running buffer alone allowed correction for binding-independent responses. Sensorgrams (time courses of the SPR signal in RU) were further corrected for non-specific binding to the anti-human PTX3 antibody (as recorded in the reference channel) and normalized to a baseline value of zero.

**AP C3 Convertase (C3bBb) and Membrane Attack Complex (MAC) Deposition Assays**

Nunc-Immuno MaxiSorp 96 well plates (Corning, USA) were coated overnight at 4°C with 10 µg/mL rhPTX3 or 3 µg/mL C3b in PBS, blocked with PBS containing 1% (w/v) BSA and 0.1% (v/v) Tween-20 for 2 h at 37°C, and washed with washing buffer (PBS containing 0.1% Tween-20 and 5 mM MgCl_2_). PTX3/C3b and PTX3/(C3b+FH) complexes were formed by incubating PTX3-coated wells at 37°C for 1h with C3b (20 µg/mL) and mixtures of C3b (20 µg/mL) and FH (10 µg/mL) in Veronal Buffer (VB, Lonza, USA), respectively. C3b/PTX3, C3b/FH and C3b/(PTX3+FH) complexes were formed by incubating C3b-coated wells at 37°C for 1h with PTX3 (20 µg/mL), FH (10 µg/mL), and mixtures of both in Veronal Buffer, respectively. After washing, plates were incubated with 10% (v/v) FH-depleted human serum (FHDHS, Complement Technology, USA) in VB for 30 min at 37°C. For C3bBb detection, surface-bound protein complexes were detached from wells by incubation with a solution of 1% (w/v) SDS and 10 mM EDTA for 1h at room temperature, run on 8-18% SDS-PAGE gels, and transferred onto PVDF membranes (Amersham WB system, GE Healthcare, USA). Protein bands were revealed with a goat anti-human CFB polyclonal antibody (1 in 2,000 dilution) followed by a Cy3-conjugated donkey anti-goat IgG polyclonal antibody (1 in 2,000 dilution, Jackson Laboratories, USA). C3bBb formation was assessed based on the appearance of a reactive band at 60 kDa (corresponding to the factor B proteolytic fragment Bb). Intensity of the Bb bands was quantitated by densitometry using the Amersham WB system Evaluation software (GE Healthcare). C3b-coated and uncoated (i.e., PBS) wells were taken as positive and negative controls, respectively, for C3bBb formation in the applied experimental conditions (i.e., in the presence of FHDHS human serum).

In another set of experiments, after incubation with 10% FHDHS as described above, wells were washed and incubated for 1h at 37°C with a rabbit anti-human sC5b-9 (MAC) polyclonal antibody (1 in 2,000 dilution, Complement Technology), followed by incubation with an HRP-conjugated goat anti rabbit IgG polyclonal antibody (1 in 5,000 dilutions, GE Healthcare) for 1h at 37°C. Chemiluminescence signals were generated and recorded as described above.

**Complement Deposition on ARPE-19 Cells**

ARPE-19 cells were cultured and stimulated as described above, then retrieved from plates by treatment with Accutase, washed with Veronal Buffer (VB), and incubated with 10 µg/mL rhPTX3 in VB or buffer alone. Cells were then challenged with 10% (v/v) normal human serum (NHS, Complement Technology) at 37°C for 30 min. Deposition of C3 (whole C3 and its proteolytic fragments, including C3b) was revealed using a goat anti-human C3 polyclonal antibody (1 in 1,000 dilution, Merck Millipore), followed by an Alexa 488-conjugated donkey anti-goat IgG antibody. Formation of MAC was assessed using a rabbit anti-human sC5b-9 plyclonal antibody (1 in 2,000 dilution, Complement Technology), followed by an Alexa 647-conjugated donkey anti-rabbit IgG polyclonal antibody. In all cases, cells were fixed with 1% (w/v) PFA in PBS, resuspended with 200 µL of a 0.2% (w/v) BSA solution in PBS^+/+^ (PBS containing 0.90 mM CaCl_2_ and 0.50 mM MgCl_2_), and analyzed by Flow Cytometry on a FACSCanto II system. Raw data were processed using the FlowJo software.

**Statistical analyses**

All data were analyzed using non-parametric tests: Mann-Whitney test (for comparison of two groups) or Kruskall-Wallis test (when comparing more than two groups), followed by Dunn’s multiple comparison correction test, as indicated in the figure legends. For each comparison p values are reported in the figures. Results are presented as mean ± standard error of the mean (SEM), and individual data points are also shown. Values of p < 0.05 were considered statistically significant. Statistical analyses were performed using the Prism 8.0 Software (GraphPad Software, USA).

**Supplementary References**

Angi, M., Kalirai, H., Coupland, S.E., Damato, B.E., Semeraro, F., and Romano, M.R. (2012). Proteomic analyses of the vitreous humour. *Mediators Inflamm* 2012**,** 148039.

Baranova, N.S., Inforzato, A., Briggs, D.C., Tilakaratna, V., Enghild, J.J., Thakar, D., Milner, C.M., Day, A.J., Richter, R.P. (2014). Incorporation of pentraxin 3 into hyaluronan matrices is tightly regulated and promotes matrix cross-linking. J Biol Chem 289, 30481-98.

Bottazzi, B., Vouret-Craviari, V., Bastone, A., De Gioia, L., Matteucci, C., Peri, G., Spreafico, F., Pausa, M., D'ettorre, C., Gianazza, E., Tagliabue, A., Salmona, M., Tedesco, F., Introna, M., and Mantovani, A. (1997). Multimer formation and ligand recognition by the long pentraxin PTX3. Similarities and differences with the short pentraxins C-reactive protein and serum amyloid P component. *J Biol Chem* 272**,** 32817-32823.

Bravman, T., Bronner, V., Lavie, K., Notcovich, A., Papalia, G.A., and Myszka, D.G. (2006). Exploring "one-shot" kinetics and small molecule analysis using the ProteOn XPR36 array biosensor. *Anal Biochem* 358**,** 281-288.

Canovi, M., Lucchetti, J., Stravalaci, M., Valentino, S., Bottazzi, B., Salmona, M., Bastone, A., and Gobbi, M. (2014). A new surface plasmon resonance-based immunoassay for rapid, reproducible and sensitive quantification of pentraxin-3 in human plasma. *Sensors (Basel)* 14**,** 10864-10875.

Livak, K.J., and Schmittgen, T.D. (2001). Analysis of relative gene expression data using real-time quantitative PCR and the 2(-Delta Delta C(T)) Method. *Methods* 25**,** 402-408.

**Table S1. Sequences of the primers used for reverse transcription quantitative polymerase chain reactions**

| **Gene symbol** | **Primer name and sequence** |
| --- | --- |
| *C3* | F: 5’-ACGCCTGGGCCGTGAAGGAGT-3’ |
|  | R: 5’-CCGCAGCCCGAGGGGGTCACA-3’ |
| *CFB* | F: 5’-GCGTGGCTCCCAGCGGCGAA-3’ |
|  | R: 5’-CGCTTCTGTTGTTCCCCTGGGCCGT-3’ |
| *CFH* | F: 5’-CCCAACTTCCCCAGTGTGTGGCAAT-3’ |
|  | R: 5’-TGGGAATCTGAGGTGGAGGTGGGCA-3’ |
| *CD46 (MCP)* | F: 5’-ACCAACATTTGAAGCTATGGAGC-3’ |
|  | R: 5’-GCCATGTATGATTCCGATCACAA-3’ |
| *CD55 (DAF)* | F: 5’-AGGCCGTACAAGTTTTCCCG-3’ |
|  | R: 5’-CCTTCTCGCCAGGAATTTTCAC-3’ |
| *CD59 (MAC-IP)* | F: 5’-CAGTGCTACAACTGTCCTAACC-3’ |
|  | R: 5’-TGAGACACGCATCAAAATCAGAT-3’ |

**Table S2. Donors used for quantitation of PTX3 in the vitreous humor**

| **ETR number** | **Age** | **Sex** | **Post-mortem time *(hrs)*** | **AMD status** |
| --- | --- | --- | --- | --- |
| ETR16 | 78 | Female | 32 | Early AMD |
| ETR17 | 82 | Male | 45 | Non-AMD |
| ETR19 | 79 | Male | 45 | Non-AMD |
| ETR26 | 82 | Female | 37 | Early AMD |
| ETR37 | 75 | Male | 47 | Non-AMD |
| ETR50 | 74 | Male | 43 | Early AMD |
| ETR51 | 78 | Female | 39 | Non-AMD |
| ETR62 | 76 | Male | 34 | Early AMD |
| ETR68 | 80 | Male | 37 | Early AMD |
| ETR76 | 77 | Female | 44 | Non-AMD |
| ETR78 | 81 | Female | 39 | Early AMD |
| ETR81 | 81 | Female | 48 | Non-AMD |

Listed are the Manchester Eye Tissue Repository (ETR) numbers of the donors used for quantitation of PTX3 in the vitreous humor, along with their age, sex, AMD status and time post-mortem when vitreous humor was withdrawn (6 non-AMD, average age = 79; 6 early AMD, average age = 79).

**
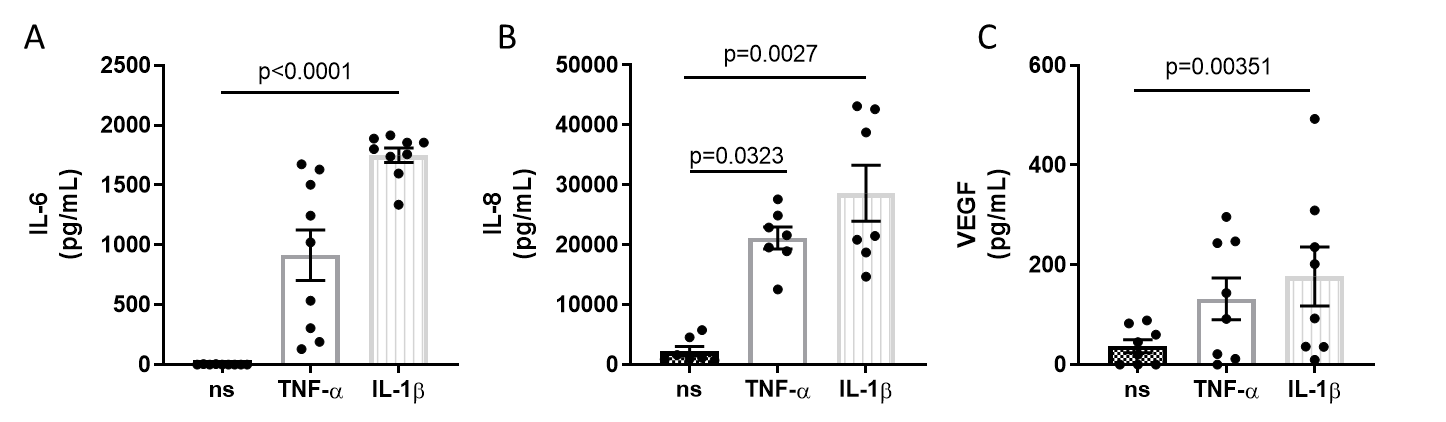
**

**Figure S1**. **TNF-α and IL-1β induce a pro-inflammatory and pro-angiogenic state in ARPE-19 cells.** ARPE-19 cells were treated with 10 ng/mL TNF-α, 10 ng/mL IL-1β, or vehicle alone for 24 h. The concentration of secreted IL-6, IL-8 and VEGF in the conditioned media was measured by ELISA. Data are expressed as mean ± SEM, n=7-9 (Kruskall-Wallis test followed by Dunn’s multiple comparison test).

**Figure S2. Effect of TNF-α and IL-1β on the binding of PTX3 to ARPE-19 cells.** ARPE-19 cells were stimulated with 10 ng/mL TNF-α, 10 ng/mL IL-1β, or vehicle alone for 24 h, then incubated with 10 µg/mL rhPTX3 for 30 min at room temperature. Cell-bound PTX3 was detected by flow cytometry using a rabbit anti-human PTX3 polyclonal antibody, followed by incubation with an Alexa 647-conjugated donkey anti-rabbit IgG polyclonal antibody. A plot (event counts *vs* fluorescence intensity) is shown that is representative of seven independent experiments (see Figure 2C for the combined data).

**Figure S3. Western blotting analysis of the alternative pathway C3 convertase (C3bBb) formation *in vitro*.** Microtiter plates were coated with C3b alone (lane 4), PTX3 followed by C3b (lane 6), or PTX3 followed by a mixture of C3b and FH (lane 7), then incubated with 10% (v/v) FH-depleted human serum (FHDHS). Surface-bound proteins were separated on SDS-PAGE gels and transferred onto PVDF membranes. Intact factor B and its proteolytic fragment Bb (indicative of C3bBb formation) were revealed as immune-reactive bands at apparent molecular weights of 93 and 60 kDa, respectively, using a goat anti-human FB polyclonal antibody. Lanes 3 and 5 refer to PBS- and PTX3-coated wells, respectively. 2 ng of purified FB and Bb (lane 1 and 2, respectively) were run as controls. Molecular weights of the applied protein ladders are indicated. A gel is shown that is representative of four generated in as many independent experiments and analyzed by densitometry (see Figure 3C for the combined data).

**Figure S4. Interaction of fluid phase PTX3 with C3b in the presence and absence of FH, and its effect on formation of C3bBb. A**) C3b-coated wells were incubated with the indicated concentrations of PTX3 or mixtures of PTX3 and FH (at [PTX3]:[FH] molar ratios of 2:1). Bound PTX3 was detected by ELISA, and data are presented as mean ± SEM from two independent experiments performed in triplicate (n=6). **B**) Microtiter plates were coated with C3b alone (lane 2), C3b followed by FH (lane 3) or PTX3 (lane 4), PTX3 followed by a mixture of C3b and FH (lane 5), or left uncoated (i.e., PBS; lane 1), then incubated with FH-depleted human serum. Surface-bound proteins were analyzed by western blotting. Intact factor B and its proteolytic fragment Bb (indicative of C3bBb formation) were revealed as immune-reactive bands at apparent molecular weights of 93 and 60 kDa, respectively, using a goat anti-human FB polyclonal antibody. 1 ng of purified factor B and Bb (lane 6 and 7, respectively) were run as controls. Molecular weights of the applied protein ladders are indicated. A gel is shown that is representative of four generated in as many independent experiments. **C**) Band intensity of the Bb species shown in **B** was measured by densitometry, and combined results are expressed as mean ± SEM, n=4.
